# Reliability of diagnostic ultrasound in measuring the multifidus muscle

**DOI:** 10.1186/s12998-015-0059-6

**Published:** 2015-04-15

**Authors:** Eirik Johan Skeie, Jan Arve Borge, Charlotte Leboeuf-Yde, Jenni Bolton, Niels Wedderkopp

**Affiliations:** MChiro, MSc, Ulriksdal 2, 5009 Bergen, Norway; DC, MSc, Ulriksdal 2, 5009 Bergen, Norway; Department Spincenter of Southern Denmark Hospital Lillebælt, Østre Hougvej 55, DK-5500 Middelfart, Denmark; Anglo European College of Chiropractic. Research Department, 13-15 Parkwood Road, Bournemouth BH5 2DF England, UK; Orthopaedic Department, Center for Spine Surgery, Hospital of Lillebaelt, Institute of Regional Health Service Research and Center for Research in Childhood Health, University of Southern Denmark, Østre Hougvej 55, DK5500 Middelfart, Denmark

**Keywords:** Diagnostic ultrasound, Measurement, Lumbar multifidus, Agreement, Reliability, Limits of agreement, Intraclass correlation coefficient

## Abstract

**Background:**

Ultrasound is frequently used to measure activity in the lumbar multifidus muscle (LMM). However previous reliability studies on diagnostic ultrasound and LMM have included a limited number of subjects and few have used Bland-Altman’s Limits of Agreement (LOA). Further one does not know if activity affects the subjects’ ability to contract the LMM.

**Methods:**

From January 2012 to December 2012 an inter- and intra-examiner reliability study was carried out in a clinical setting. It consisted of a total of four experiments with 30 subjects in each study. Two experienced examiners performed all measurements. Ultrasound measurements were made of: 1. the LMM in the resting state, 2. during a contracted state, 3. on subsequent days, and, before and after walking. Reliability and agreement was tested for 1. resting LMM, 2. contracted LMM, and 3. thickness change in the LMM. Mean values of three measurements were used for statistical analysis for each spinal level. The intra-class correlation coefficient (ICC) 3.1 and 3.2 was used to test for reliability, and Bland-Altman’s LOA method to test for agreement.

**Results:**

All of the studies indicate high levels of reliability, but as the LMM thickness increased (increasing contraction) the agreement between examiners was poorer than for low levels of contraction.

**Conclusions:**

The use of diagnostic ultrasound to measure the LMM seems to be reliable in subjects who have little or no change in thickness of the LMM with contraction.

## Introduction

### The lumbar multifidus muscle and low back pain

It is well known that non-specific low back pain (LBP) is a prevalent disorder often with numerous recurring episodes [1]. Currently there is no objective clinical test that is able to differentiate subjects with nonspecific LBP from pain free subjects, nor is there any clinical test than can predict the occurrence or recurrence of LBP. Even though the exact cause of LBP remains unknown, some studies indicate that fat infiltrations in the multifidus musculature (LMM) are associated with back pain [2]. Numerous studies have been carried out on the LMM in relation to the presence of LBP with and without radiculopathy [3-9], as well as LMM size and function as a prognostic factor for LBP [10,11], predictive effects of changes in the LMM in LBP patients [12,13] and LMM changes in relation to treatment of LBP [14-16]. Changes of the LMM function have also been noted in people who previously had LBP [17] and even in those with experimentally induced LBP [18]. Therefore it seems possible that there may be a link between the function and/or morphology of the LMM and LBP. Hence function of the LMM may be easily altered by pain and slow to recover.

### Evaluating the LMM with diagnostic ultrasound

When evaluating the LMM with ultrasound, this is done by comparing the thickness of resting muscle with that of activated muscle. The reason for this is findings in prior studies that have demonstrated reduced ability to contract the LMM in low back pain patients [7,9] as well as in patients who have previously suffered from LBP [17]. Hodges et al. [19] investigated the use of ultrasound to measure muscle contraction on several muscles other than the LMM. The study found the architectural parameters measured by ultrasound and EMG showed a nonlinear relationship, and the majority of muscle thickness change took place in the range up to 30% of maximal voluntary contraction [19]. For the LMM a close correlation was found between values measured by ultrasound and activity measured by EMG when the contractions were in the range of 19 to 34% of maximum contraction [20].

Earlier studies on diagnostic ultrasound and the LMM differed greatly on methodology, procedures, equipment, muscles tested, sample size, LBP presentation, and levels of physical fitness of participants. A systematic review by Hebert et al. [21] reported poor methodological quality of previous studies on diagnostic ultrasound and LMM, only 6 of the 24 studies included in the systematic review were considered high quality studies.

When measuring the thickness of the LMM, earlier studies have shown that averaging the thickness of three measurements optimizes reproducibility [22,23]. Very good inter-rater agreements between novice and experienced examiners have been found when measuring LMM thickness [24]. Good inter- and intra-rater reliability has also been reported between experienced examiners [25] and novice examiners [23,26,27]. In order to activate the LMM one can lift either the contralateral arm or leg. An earlier study found only marginal difference in contraction when lifting the contralateral arm or leg: The same study also noted that transducer position has little effect on intra and inter-rater reliability of diagnostic ultrasound and the LMM [23]. The systematic review by Hebert et al. [21] highlights that reliability increases with more experienced examiners, and that only a minority of studies have reported low levels of reliability.

### Need for further studies on diagnostic ultrasound

Criticism has been raised against several of the studies on inter- and intra-rater reliability of the LMM when measured with diagnostic ultrasound. Hebert et al. [21] highlighted different methods in measuring the LMM in previous studies, and several of these had small sample sizes (<15), asymptomatic subjects, and only some of the studies looked at the measurement of contraction. None of the previous studies investigated how general activity, such as gait might affect measurements of the LMM using diagnostic ultrasound. The reason for investigating gait, is the suggestion that the spine is the key to locomotion of the lower limbs [28]. More recent studies have shown increased electromyographic activity in the LMM during walking [29].

### Methodological considerations

Previous studies that investigated reproducibility of measurements of LMM with diagnostic ultrasound have done so by examining reliability of measurements. To test this statistically, the intra-class correlation coefficient (ICC) is commonly used. However, the concept of reproducibility consists also of agreement. Agreement is best illustrated with Bland-Altman’s Limits of Agreement (LOA) method [30-33] because it helps detect any systematic differences between the individual measurements (i.e., fixed bias) and is able to identify possible outliers. However only rarely in previous studies on diagnostic ultrasound and the LMM have both these methods been used [26].

### Aim and objectives of the present study

In order to bring forth a coherent picture on the issue of the potential usefulness of ultrasound diagnosis on the LMM in people with LBP, a number of projects were carried out. We started with the most basic aspects, moving towards the more advanced ones, using both the ICC and LOA methods for our statistical analyses. Specifically, the study had the four following objectives in relation to the ultrasound diagnostic procedure on the LMM:

1. To study the inter-examiner reliability of diagnostic ultrasound when measuring LMM thickness on one still image.
2. To study the inter-examiner reliability of diagnostic ultrasound when measuring LMM contraction on two sets of still images.
3. To study the intra-examiner reliability of diagnostic ultrasound when measuring LMM contraction on two different occasions.
4. To study the stability of measurements of LMM contraction with diagnostic ultrasound by comparing these before and after the subjects exercised.

## Methods

### Examiners

Inter and intra-examiner reliability was tested between two chiropractors who were both experienced in diagnostic ultrasound for the musculoskeletal system. Examiner 1 had four years of experience in diagnostic ultrasound and examiner 2 had eight years of experience. At the time of the study both the examiners held a postgraduate diploma in diagnostic ultrasound. Before the study, both examiners agreed upon and developed the protocol of diagnostic ultrasound that was applied in this study.

### Study subjects

An a priori decision was made to include 30 study subjects to test each of the four study objectives. These subjects were recruited consecutively from a chiropractic practice from January 2012 to December 2012. The sample size was considered a convenience sample as the study was conducted in a routine clinical practice setting. The majority of these subjects were LBP patients although patients with other spinal complaints such as mid back pain, neck pain, and/or extremity pain were also included. In addition some pain-free subjects were recruited from outside the clinic. This case mix was to include subjects with the potential ability to produce a contraction of the LMM as well as those with the potential not to. Subjects were recruited during the clinic’s opening hours, normally around the end of the day and during lunch hours when both examiners were available. Each of the total 120 subjects took part in only one of the projects outlined above. All subjects gave verbal and written consent to inclusion in the study. Application for ethics approval was sent to the Regional Committees for Medical and Health Research Ethics (REC) in Norway. REC considered the project a quality assurance project and therefore no special permission from REC was needed to complete the project.

### Procedures

#### Ultrasound measurements

**Figure 1:**
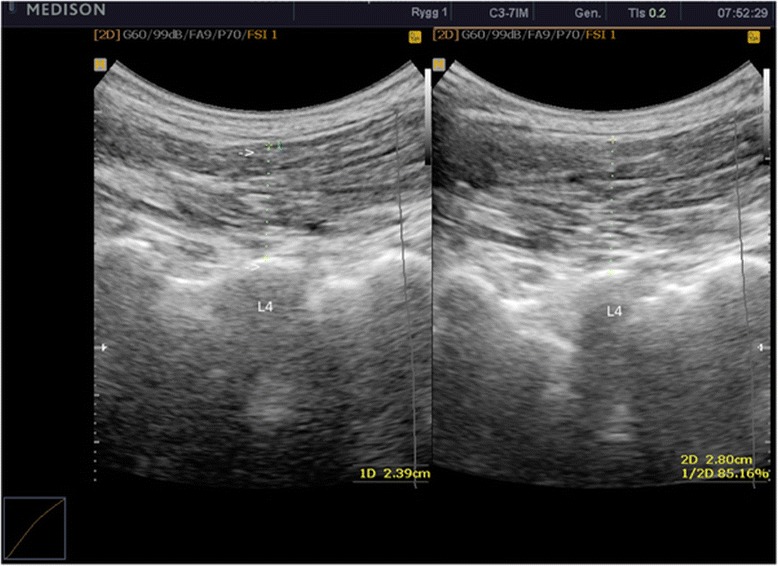
**Ultrasound image of resting LMM (left image) contracted LMM (right image).** Calipers placed on the apex of facet joint of L4, and on the interface between the thoracolumbar fascia and subcutaneous fat.

In this study all the measurements of the LMM were taken with the subjects in a prone position with a pillow placed under the abdomen to flatten the lumbar lordosis as this provides better contact for the transducer. A Medison Accuvix V10 ultrasound scanner with a 3–7 MHz curvilinear probe was used. To identify the level of the LMM in the lumbar spine, the transducer was placed longitudinally along the spine with the midpoint over the spinous processes of interest. The sacrum was recognized as a longitudinal structure in contrast to the shorter curved spinous processes. The probe was then moved laterally and angled slightly medially until the facet joint in question could be visualized as described by Kiesel [20]. At this point the probe was directly overlying the LMM, and a measurement was taken from the apex of the facet joint to the plane between the thoracolumbar fascia and the subcutaneous fat. The reason for utilizing the on-screen callipers was to make the study as clinically relevant as possible. Previous studies have analysed the images offline. However, this is not common in a clinical setting. Care was taken not to move too far laterally as this would lead to imaging of the erector spinae muscles and not the LMM. Figure 1 illustrates placement of the calipers.

### Objective 1: Inter-examiner reliability of LMM thickness on the same still image

For all study subjects in objective 1, a single image was generated of the LMM by one of the examiners. The first examiner then placed a marker on the image on the mammillary process of the level to be measured. Examiner 1 subsequently measured the distance three times with the calliper software on the ultrasound machine, saving each image onto the ultrasound machine’s hard drive. The callipers and saved images were removed before examiner 2 entered the room, leaving only the still image with the marker in place on the screen. Examiner 2 then performed the same measurement procedure. Thereafter the data were transferred to a separate paper by examiner 1 who calculated mean values.

### Objective 2: Inter-examiner reliability of LMM contraction on separate still images

For all subjects, images of the LMM in the resting and contracted states were generated independently by each of the examiners. The spinal level to be measured was chosen from predetermined criteria (a total of thirty average measurements, fifteen from the left and fifteen from the right, and evenly distributed between L3-L5). Examiner 1 generated an image of the LMM in the resting state with the subject in prone position (Figure 1). Thereafter a split screen was utilized and the subject performed the contralateral arm lifting task as described by Kiesel [20] but with no hand held load. Then a second image (Figure 1: Image 2) was captured of the contracted LMM with the arm in the elevated position, and the thickness of the LMM was measured on screen of the two images (Figure 1: Image 1: resting thickness, Figure 1: Image 2: contracted thickness). This procedure was performed three times by both examiners for each subject, giving three sets of measurements of the LMM in the resting and contracted states for each level for each examiner. The three sets of images with the measurements in place were saved onto the ultrasound machine’s hard drive. Examiner 1 removed the saved images from the screen before examiner 2 entered the room. Examiner 2 then repeated the same procedure. After examiner 2 left the room, the data were then transferred to two separate sheets of paper by examiner 1. Examiner 1 calculated mean measurements for the individual measurements by both examiners (mean resting and contraction values). In addition contraction of the LMM was expressed as raw change in thickness (contracted LMM minus resting LMM). Contraction was expressed as an exact change in thickness and not in a relative percentage because there is missing evidence to support that the LMM contracts as a unit.

### Objective 3: Intra-examiner reliability of LMM contraction using two sets of still images on two different days

For all subjects, three sets of measurements were generated on two different days giving a total of six sets of measurements per subject. Examiner 1 performed all measurements. To reduce the risk of recall, a minimum of five days elapsed between measurements during which a large number of patients had been examined, making recall of previous measurements unlikely. The procedure for obtaining the images was the same as for objective 2. The measurements obtained by the examiner were saved onto the ultrasound machine, and recorded on two different sets of paper that were kept separate until all measurements had been obtained. The first sets of measurements were deleted off the ultrasound machines hard drive on the same day as they were generated. This was done to avoid examiner 1 being able to read the first set of measurements when performing measurements on the second day. Examiner 2 then calculated the mean of resting and contracting LMM values for day 1 and day 2.

### Objective 4: Repeatability of measurements of LMM contraction with diagnostic ultrasound before and after the subjects walked around the table

For all subjects examiner 1 generated two sets of images. Again, examiner 1 performed all measurements. The procedure for obtaining resting and contraction measurements of LMM were the same as in objectives 2 and 3. For each subject three sets of measurements were taken both before and after the subject walked around the table (exercised). When recording the measurements, examiner 1 first saved the first three sets of measurements on the ultrasound machine’s hard drive, after which the subject exercised. During the exercise the first sets of measurements had been cleared from the screen. The second three sets of measurements taken were saved on the same subject file but annotated as “after”. The reason for clearing the images from the screen was to prevent examiner 1 from reading the measurements from the “before” measurements when recording the second sets of measurements. After the measurements were completed, examiner 2 transferred the data onto a separate sheet of paper and calculated mean values for the individual measurements by examiner 1 (mean resting thickness and contraction thickness before the patient had walked, and mean resting and contraction values after the subject had walked around the table). The contraction was expressed as raw change in thickness (contracted LMM – resting LMM).

### Statistical analyses

Correlation between examiners was measured in three ways:

1. For study objectives 1 to 4, ICC were determined in two ways, both as two way mixed single measures (3.1) and as two way mixed average measures (3.2) in order to evaluate inter- and intra-rater reliability. ICC 3.1 and 3.2 are the correct forms of ICC to use when the subjects are randomly selected but the examiners are not [34]. In this analysis, both subjects and examiners are seen as potential sources of systematic variability.

There is no consensus of what constitutes a good ICC value [35]. According to the guidelines by Kottner et al. [33] the ICC values should be at least 0.90 or 0.95 if individual and important decisions should be made based on ICC statistics. A systematic review by Hebert et al. [21] on the reliability of diagnostic ultrasound on the abdominal and lumbar trunk muscles used ICC values above 0.75 to indicate good reliability and below 0.75 to indicate poor reliability.

2. LOA were also calculated for study objectives 1, 2 and 4 and shown in order to determine differences between the means of the measurements. The LOA is shown as a graph in which the individual measurements are plotted making it possible to observe if the results vary as a function of the size of the measurements.

3. In addition to the ICC values for study objective 3, a linear plot was constructed in order to evaluate the level of LMM contraction in the subjects on two different days.

The analyses were carried out by an independent person (NW) using STATA version 12.1.

## Results

### Descriptive data

A detailed description of the study subjects is shown in Table 1. Each experiment consisted of a different sample of 30 subjects.

**Table 1:** **Descriptive data on subjects**

**Subjects, total.**									
**Total (N)**	**Male (N)**	**Female (N)**	**Mean age (Yrs.)**	**Age range (Yrs.)**	**SD (Yrs.)**	**LBP (N)**	**Neck/Midback pain (N)**	**Extremity pain (N)**	**Pain free (N)**
**120**	64	56	38	20-69	±12	88	23	4	5
**Study objective 1**									
**30**	18	12	38	20-69	±13	25	5	0	0
**Study objective 2**									
**30**	14	16	37	20-65	±12	20	5	1	4
**Study objective 3**									
**30**	15	15	38	20-59	±11	23	7	0	0
**Study objective 4**									
**30**	17	13	40	20-68	±11	20	6	3	1

### Objective 1. To study the inter-examiner reliability of diagnostic ultrasound when measuring LMM thickness on one still image

**Figure 2:**
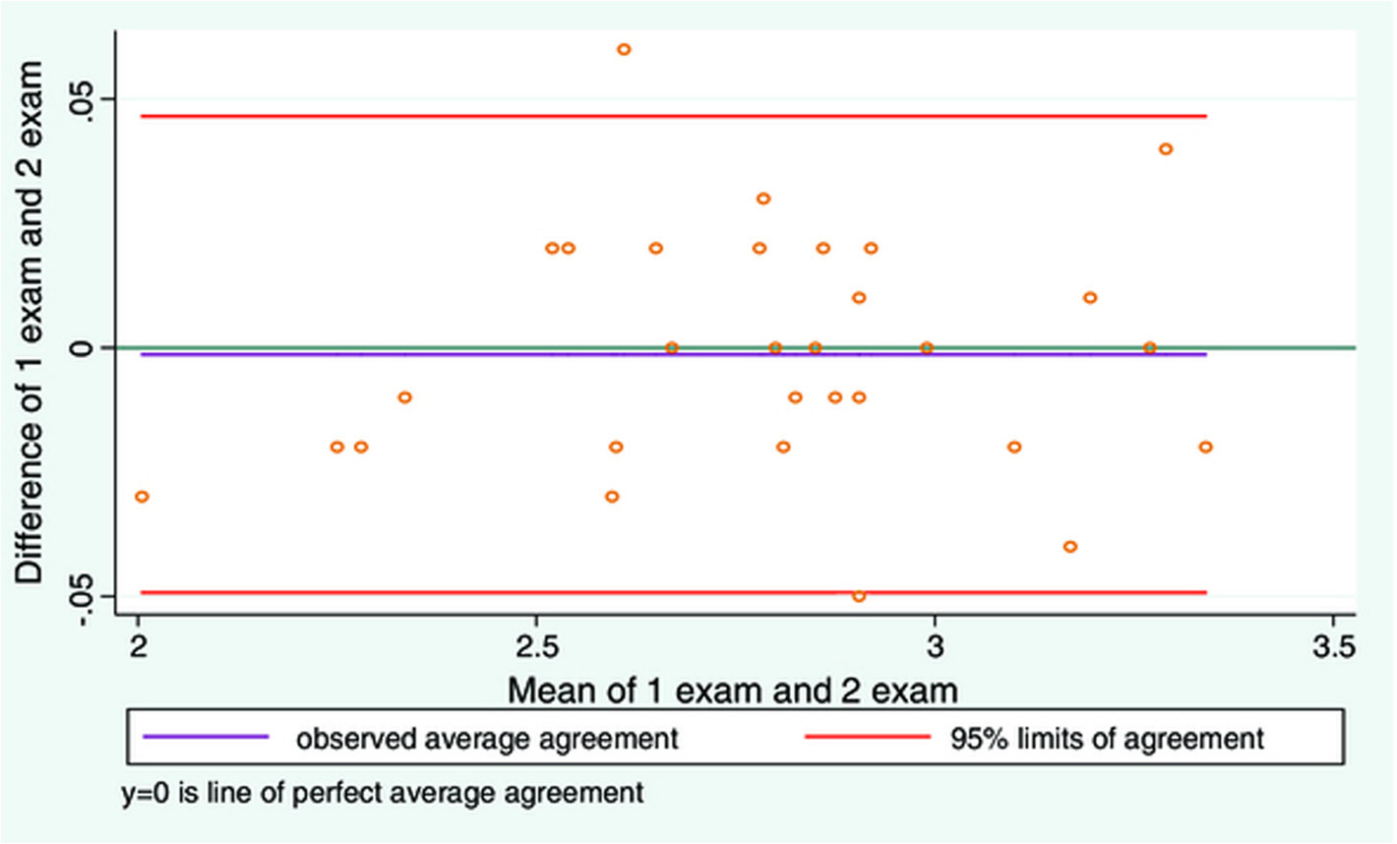
**LOA plot showing agreement between examiner 1 and examiner 2.** Study objective 1, measurement of LMM thickness on one still image (N = 30).

Good inter-examiner reliability was found between examiners (Table 2). The mean difference between examiners was low and the LOA narrow in range (Figure 2, Table 3). The greatest difference on an individual measurement between the two examiners, gave a measurement difference of approximately 2% when applied to the average LMM thickness.

**Table 2:** **Mean measurements for LMM and ICC values for study objective 1–4**

**Objective 1 Interexaminer reliability of measuring LMM thickness using one still image**			
Mean LLM thickness examiner 1	Mean LLM thickness examiner 2	ICC average	ICC individual
27.9 mm ± 3.2 mm	27.9 mm ± 3.2 mm	0.999 (0.997-0.999)	0.997 (0.994-0.999)
**Objective 2 Interexaminer reliability of measuring LMM contraction using two sets of still images.**			
Mean relaxed LLM thickness examiner 1 (distance 1)	Mean relaxed LLM thickness examiner 2 (distance 1)	ICC average	ICC individual
28.9 mm ± 6.4 mm	29.0 mm ± 6.1 mm	0.97 (0.94-0.99)	0.95 (0.89-0.98)
Mean contracted LLM thickness examiner 1 (distance 2)	Mean contracted LLM thickness examiner 2 (distance 2)	ICC average	ICC individual
32.1 mm ± 7.0 mm	32.0 mm ± 6.7 mm	0.97 (0.94-0.99)	0.95 (0.90-0.98)
Distance 2–1 examiner 1	Distance 2–1 examiner 1	ICC average	ICC individual
3.1 mm ± 2.2 mm	3.0 mm ± 2.0 mm	0.98 (0.96-0.99)	0.97 (0.92-0.98)
**Objective 3 Intraexaminer reliabilty of measuring LMM contraction using 2 sets of still images taken on 2 different days.**			
Mean relaxed LLM thickness (distance 1 day 1)	Mean relaxed LLM thickness (distance 1 day 2)	ICC average	ICC individual
28.4 mm ± 5.3 mm	28.4 mm ± 4.8 mm	0.99 (0.97-0.99)	0.97 (0.94-0-99)
Mean contracted LLM thickness (distance 2 day 1)	Mean contracted LLM thickness (distance 2 day 2)	ICC average	ICC individual
29.7 mm ± 6.0 mm	29.6 mm ± 5.5 mm	0.97 (0.93-0.99)	0.94 (0.88-0.97)
Distance 2–1 day 1	Distance 2–1 day 2	ICC average	ICC individual
1.4 mm ± 1.7 mm	1.3 mm ± 1.7 mm	0.97 (0.93-0.99)	0.94 (0.88-0.97)
**Objective 4 Measuring LMM contraction before and after a motor task on two sets of still images.**			
Mean relaxed LLM thickness (distance 1 before task)	Mean contracted LLM thickness (distance 2 before task)	Mean relaxed LLM thickness (distance 1 after task)	Mean contracted LLM thickness (distance 2 after task)
30.6 mm ± 5.5 mm	34.1 mm ± 6.6 mm	29.9 mm ± 5.3 mm	34.6 mm ± 6.4 mm
Distance 2–1 before	Distance 2–1 after	ICC average	ICC individual
3.5 mm ± 2.6 mm	3.5 mm ± 2.5 mm	0.98 (0.97-0.99)	0.97 (0.94-0.99)

**Table 3:** **Mean difference and LOA range study 1, 2, and 4**

**Objective 1**		
	**Mean difference**	**LOA range**
Relaxed LMM	0.01 mm ± 0.24 mm	[−0.48; 0.47 mm]
Objective 2		
Relaxed LMM	0.08 mm ± 2.0 mm	[−4.07; 3.92 mm]
Contracted LMM	0.06 mm ±2.0 mm	[−3.93; 4.06 mm]
Contracted-Relaxed LMM	0.14 mm ±0.55 mm	[−0.94; 1.22 mm]
Objective 4		
Relaxed LMM	0.7 mm ± 0.9 mm	[−1.09; 2.49 mm]
Contracted LMM	0.7 mm ± 0.9 mm	[−1.18; 2.51 mm]
Contracted-Relaxed LMM	0.04 mm ± 0.65 mm	[−1.32; 1.25 mm]

### Objective 2. To study the inter-examiner reliability of diagnostic ultrasound when measuring LMM contraction on two sets of still images

**Figure 3:**
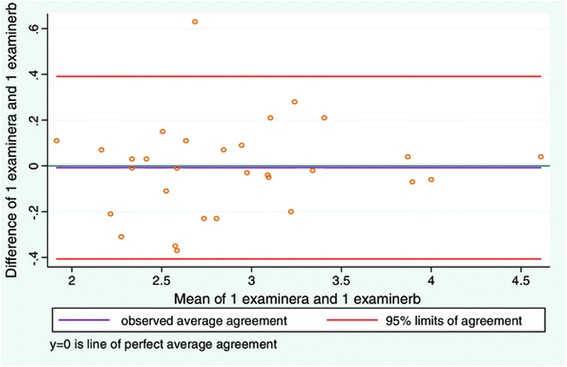
**LOA plot showing agreement between examiner 1 and examiner 2.** Study objective 2, measurement of resting LMM on two sets of images (N = 30).

**Figure 4:**
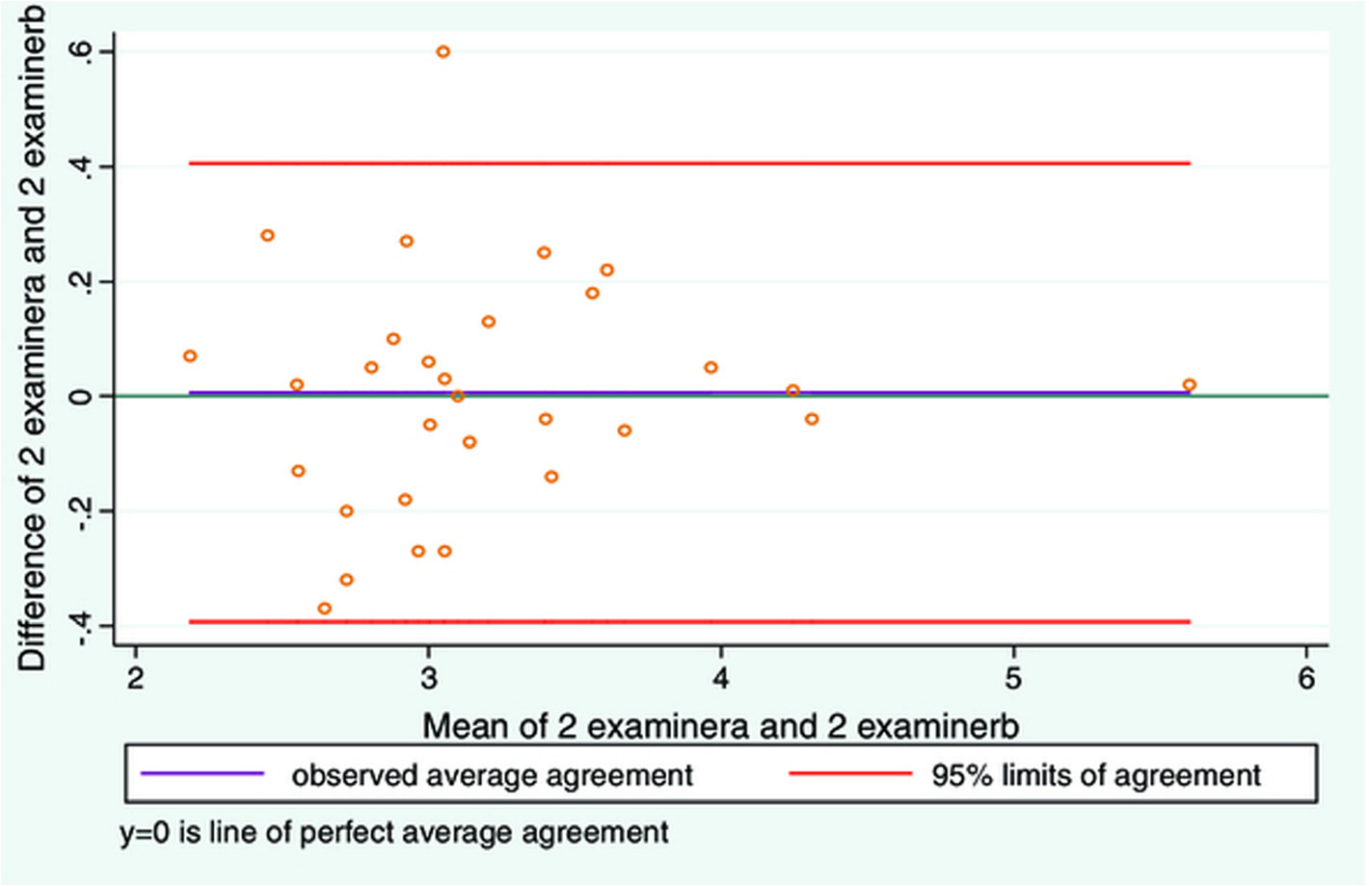
**LOA plot showing agreement between examiner 1 and examiner 2.** Study objective 2, measurement of contracted LMM on two sets of images (N = 30).

Good inter-examiner reliability was also found between examiners when measuring resting and contracted LMM (Table 2). The LOA plots (Figures 3 and 4, and Table 3) for resting and contracted LMM showed a small average difference between examiner 1 and 2. However the LOA plots (Figures 3 and 4, and Table 3) were substantially wider than in study 1. The average difference between examiners measuring resting LMM was very low (Table 3), but the greatest difference on an individual measurement equated to a difference of as much as 21% between examiners (Figure 3). For the contracted LMM the average difference between examiners measuring resting LMM was very low (Table 3). But the greatest difference on an individual measurement of the LMM resulted in a 19% difference between examiners (Figure 4).

**Figure 5:**
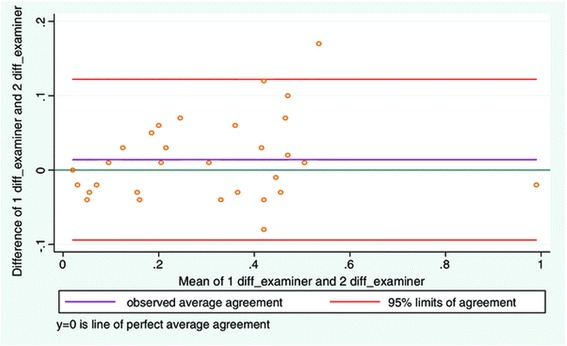
**LOA plot showing agreement between examiner 1 and examiner 2.** Study objective 2, measurement of contraction (distance 2 – distance 1) LMM on two sets of images (N = 30).

When LMM contraction was expressed as contracted LMM minus relaxed LMM good inter-examiner reliability was found (Table 2). The LOA plot (Figure 5, Table 3) demonstrated a low average difference between the examiners. But compared with the LOA plots (Figures 3 and 4) for measurements of contracted and relaxed LMM, the average difference between examiners increased when expressing contraction as LMM minus relaxed LMM. The greatest difference on an individual measurement equated to a 45% difference in measurements between the two examiners. The LOA (Figure 5) demonstrated a funnel shape with the opening to the right. On the x-axis the volume increased towards the right suggesting poorer agreement with increasing muscle thickness.

**Figure 6:**
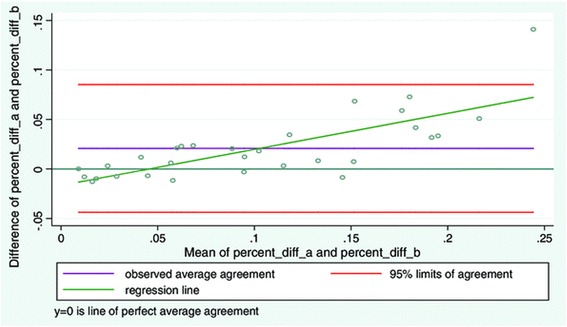
**LOA plot showing agreement between examiner 1 and examiner 2.** Study objective 2, measurement of LMM contraction expressed as relative % (distance 2 – distance 1)/distance 1) on two sets of images (N = 30).

It is also possible to express contraction as a relative percentage change and not as a raw measurement. This was performed as a separate analysis to see if it changed the LOA plot. Figure 6 shows contraction expressed this way. This resulted in a change in the funnel shape of the LOA plot into a more linear increase indicating that the examiners agreed less as the muscle thickness increased.

### Objective 3. To study the intra-examiner reliability of diagnostic ultrasound when measuring LMM contraction on two different days

Again, there was good intra-examiner reliability both for relaxed and contracted LMM (Table 2). ICC values for contraction expressed as contracted LMM minus relaxed LMM (Table 2) also demonstrated excellent intra-examiner reliability.

**Figure 7:**
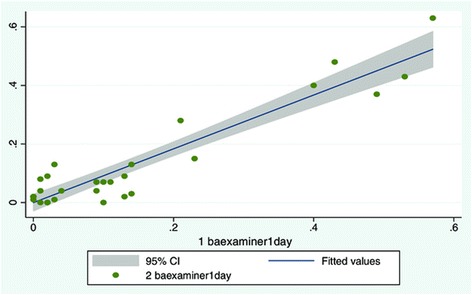
**Scatter plot of subjects in study objective 3.** Day to day scatter, x-axis shows day 1, y axis day 2.

The linear plot in Figure 7 shows little change in measurements from day to day, and that the vast majority of the subjects had little or no ability to contract their LMM. Only five subjects are seen on the right end of the scale demonstrating a volume change representing contraction. Four of the subjects had around 4 mm volume increase of the LMM and one subject had around 6 mm volume change. On average this equates to a relative thickness change between 14 and 20%. This study did not attempt to correlate the level of pain with contraction, so it is not possible to determine whether these subjects suffered from LBP.

### Objective 4. To study the repeatability of measurements of LMM contraction with diagnostic ultrasound before and after the subjects walked around the table

**Figure 8:**
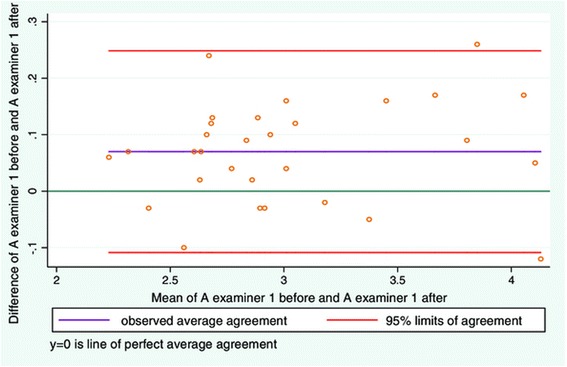
**LOA plot showing agreement between examiner 1 before and after the subject performed a motor task.** Study objective 4, measuring resting LMM before and after a simple motor task on two sets of images (N = 30).

**Figure 9:**
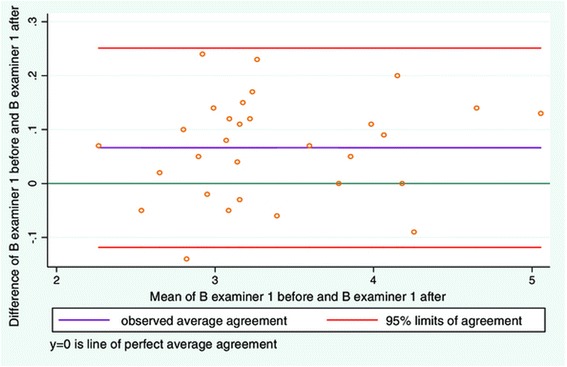
**LOA plot showing agreement between examiner 1 before and after the subject performed a motor task.** Study objective 4, measuring contracted LMM before and after a simple motor task on two sets of images (N = 30).

**Figure 10:**
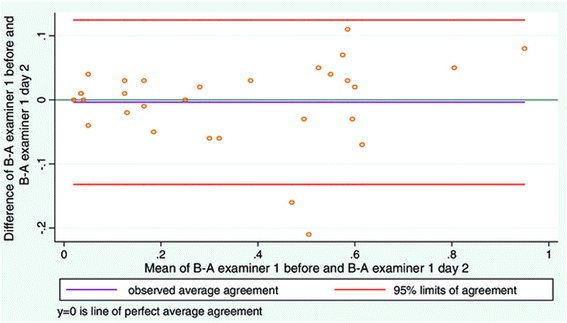
**LOA plot showing agreement between examiner 1 before and after the subject performed a motor task.** Study objective 4, measurement of contraction (distance 2 – distance 1) LMM on two sets of images (N = 30).

There was good intra-examiner reliability for relaxed and contracted LMM on days 1 and 2 (Table 2). Good intra-examiner agreement was also seen for contraction expressed as contracted minus relaxed LMM (Table 2). The LOA plots for relaxed and contracted LMM (Figures 8 and 9) were very similar to those in study objective 2 (Figures 3 and 4). The average difference for relaxed and contracted LMM was still low although greater than those found in study 2 (Table 3). Nonetheless the standard deviation for resting and contracted LMM is lower than that seen in study objective 2. The greatest difference for an individual measurement was equal to 6% measurement difference before and after the subject exercised. For contracted LMM the greatest difference on an individual measurement was equal to 5% measurement difference. When expressing contraction as (contracted LMM minus relaxed LMM) a similar plot to Figure 5 is seen in Figure 10. Again a moderate funnel shape can be seen, indicating less agreement as the LMM thickness increases. The average difference is also very low (Table 3). The greatest difference in LMM contraction on an individual measurement gave a measurement difference in muscle thickness as high as 7% before and after the subject exercised.

## Discussion

We performed four independent studies to test if diagnostic ultrasound can be used to reliably examine the thickness of the LMM in situations that relate to the various stages of examination. To analyse our data, we used both ICC and LOA. Our results were encouraging. Average measurements were used for analysis. The reliability of the measurements of LMM thickness was good in all four studies. This was the case when two examiners used the same still image, when they used two sets of still images, when one examiner measured the same person on two different days, and before/after the study-subject had walked around for a while.

However, it was noted that good agreement was mainly present in subjects who had little or no change in muscle thickness (contraction), probably making this method less reliable to measure thickness change as seen with contraction. Because this study sample consisted mainly of people with chronic back problems, it was not possible to study further the cut-points for good and less good reliability.

### Limitations and weaknesses

Another weakness was that the examiners in these four experiments were clinicians in the clinic where the study subjects were treated. This meant that they would have met and/or treated several of these subjects. Nevertheless, many patients come through this clinic over time, a large proportion of which would be examined with diagnostic ultrasound. It would be impossible for the clinicians to remember individual values to a larger extent, and none of them had a special need to “prove” anything, but performed this study with an open and curious mind. It is unlikely that the results would be biased for this reason.

The subjects in this study were recruited from a clinical setting, the majority of which had LBP. This can be seen as both a strength and a weakness. It would have been preferable with a more mixed study sample, but the presence of people with LBP made it possible to study the usefulness of diagnostic ultrasound in a typical setting. The negative aspect is that the results cannot necessarily be generalized to other populations.

### Comparison with other studies

When comparing our results to others one can only look at the ICC values. Our results, are all similar to previous studies [21-23,26,27]. The main difference from our study to others is that we have demonstrated through the LOA analysis, a poorer agreement between two examiners who measure LMM thickness on two different sets of images. We also found less agreement between two examiners who measure contraction of the LMM. The agreement does seem to diminish when the thickness of the LMM is increasing more than 4 mm (relative increase of approximately 14%).

It has previously been shown that it is difficult for subjects with LBP to contract the LMM [18]. Our study did not aim to correlate LBP and ability to contract the LMM, however the majority of the subjects were LBP sufferers and this might be the reason why the majority of subjects had little or no ability to contract the LMM. We also included subjects without LBP, which may be reflected in the measurements that indicate a thickness increase in the LMM. As we only wanted to investigate the measurements this needs to be explored further in other studies.

### Recommendations for further studies

Further exploration of utilization of diagnostic ultrasound on the LMM is needed. The examiners showed a low level of agreement when measuring LMM thickness change in the subjects who were able to contract of the LMM, but a good level of agreement when measuring LMM thickness change in the subjects who were not capable of contracting the LMM. It could be possible to categorize the contraction in groups to see if this increases the agreement. However this would be easier if one could use relative contraction measured in % as a scale. But if one were to use relative contraction as a measurement, further studies need to be conducted to see if different parts of the LMM contracts as a unit. From a more clinical perspective correlation between pain and LMM contraction measured with diagnostic ultrasound needs to be performed, as well as studies that examine subjects who never had low back pain to obtain more knowledge of how the LMM normally would contract. The clinical utilization of diagnostic ultrasound in measuring the muscle contraction of the LMM is not clear, as normal ranges are not fully established [36]. However, diagnostic ultrasound could possibly be used for identifying subjects who are not capable of contracting the LMM.

## Conclusion

Our results indicate that ultrasound examination of the lumbar multifidus muscle is a reliable method when used by experienced examiners in people with chronic LBP, with poor contracting ability of their multifidus muscles and the average of three measurements is utilized.
